# Prevalence and associated factors of overweight and obesity among schoolchildren in Hanoi, Vietnam

**DOI:** 10.1186/s12889-019-7823-9

**Published:** 2019-11-08

**Authors:** Thuy Thi Phuong Pham, Yumi Matsushita, Lien Thi Kim Dinh, Thanh Van Do, Thanh Thi The Nguyen, Anh Tuan Bui, Anh Quoc Nguyen, Hiroshi Kajio

**Affiliations:** 1 NCGM-Bach Mai Hospital Medical Collaboration Center, Hanoi, Vietnam; 2 Center for Clinical Sciences, National Center for Global Health and Medicine, Tokyo, Japan; 3 Center of Nutrition of Bach Mai Hospital, Hanoi, Vietnam; 4 International Department, Bach Mai Hospital, Hanoi, Vietnam; 5 Biochemistry Department, Bach Mai Hospital, Hanoi, Vietnam; 6 Bach Mai Hospital, Hanoi, Vietnam; 7 Department of Diabetes, Endocrinology and Metabolism, Central Hospital, National Center for Global Health and Medicine, Tokyo, Japan

**Keywords:** Overweight, Obesity, Children, Prevalence, Vietnam

## Abstract

**Background:**

The prevalence of overweight and obesity (OW/OB) has increased rapidly in Vietnam. This study aimed to elucidate the factors influencing OW/OB among secondary schoolchildren.

**Method:**

A survey was conducted in January 2014 in four randomly selected state schools in two Hanoi urban districts, and 821 students in grade six (11–12 years old) participated. Definitions of OW/OB followed the World Health Organization standard cut-offs.

**Results:**

Overall, 4.1% of children were underweight, 59.7% were normal weight, 17.1% were overweight, and 19.1% were obese. The odds of OW/OB were lowest among children whose parents had college/university degrees [father (aOR =0.65, 95% CI: 0.42–1.00); mother (aOR =0.63, 95% CI: 0.41–0.97)] compared with those whose parents had only a primary education. Children with an OW/OB family history had an increased risk of OW/OB. Other associated factors include parental OW/OB and birth weight (BW). The odds of OW/OB were highest among children with parents with OW/OB [father (aOR =2.022, 95% CI: 1.34–3.04); mother (aOR =2.83, 95% CI: 1.51–5.30)] compared with those with normal-weight parents. Children with both parents having OW/OB [both parents (aOR =6.59, 95% CI: 1.28–33.87) had the highest risk, followed by one parent (aOR =2.22, 95% CI: 1.50–3.27)] and then neither parent having OW/OB. Moreover, high-birth-weight children [BW ≥ 3500 g (aOR =1.52, 95% CI: 1.07–2.15)] had greater odds than did normal-birth-weight children.

Children who slept 11 h per day [8–11 h (aOR =0.57, 95% CI: 0.40–0.81) or more (aOR =0.44, 95% CI: 0.22–0.87)] had lower OW/OB odds than those who slept 8 h or less. Children with specific positive lifestyle behaviours had lower risk of OW/OB than those who did not engage in positive lifestyle behaviours. The odds were lower among children who exercised for weight reduction (OR = 0.16, 95% CI: 0.11–0.23), lowered food intake (aOR = 0.12, 95% CI: 0.09–0.17), and added vegetables to their diet (aOR = 0.26, 95% CI: 0.19–0.35).

**Conclusion:**

The results suggest that parents and children with OW/OB parents or a high BW should be educated to prevent OW/OB at an early stage. Positive lifestyle behaviours should be adopted by the students.

## Background

Overweight and obesity (OW/OB) are global epidemics. A report from the WHO indicated that in 2016 more than 18% of children and adolescents aged 5–19 were overweight or obese worldwide [1]. In Vietnam, the prevalence of OW/OB among children has also increased rapidly. According to the Vietnamese National Surveillance survey in 2010, the rate of OW/OB among children under 5 years old in Vietnam was 5.6% (6.5 and 4.2% for urban and rural areas, respectively). The current rate is six times higher than it was in 2000 [2]. Obesity is a risk factor for non-communicable diseases (NCDs), such as type 2 diabetes, hypertension, and coronary artery disease. In 2015, there were 3.5 million cases of diabetes, and it is estimated that approximately 53,458 deaths were attributed to diabetes in Vietnam [3]. Moreover, Vietnam was identified as a country with a high incidence of NCDs, which are responsible for approximately 73% of all deaths, accounting for more than 379,000 deaths per year. Over one-third of deaths associated with NCDs are caused by cardiovascular diseases [4, 5]. Children whose parents are both obese have an 80% risk of developing obesity during their lifetime. If only one parent is obese, the child’s risk declines to 40–50% [6, 7]. Controlling OW/OB and chronic diseases was recognized as an area of major importance by the Government of Vietnam. This is one of the six strategy areas of the National Nutrition Strategy for the period of 2011–2020 and the vision for 2030 that was signed by the Prime Minister of the Socialist Republic of Vietnam in February 2012 [8]. In Vietnam, there are several studies that examined the prevalence of OW/OB among children less than 10 years old; however, very few studies have been conducted with children 11–12 years old (grade 6). According to the National Institute of Diabetes and Digestive and Kidney Diseases, the National Institutes of Health, children in 6th grade (secondary school) are generally 12 years old and in early adolescence. This is a time of both physical and metabolic, as well as emotional and mental, growth and development. Children are assuming more responsibility for their own choices [9]. Additionally, in meta-analysis articles related to the prevalence of overweight and obesity in Asia and in the world, data about the OW/OB status of children at this age in Vietnam were not shown [10, 11]. The present study aimed to determine the prevalence of OW/OB among secondary school children in Hanoi, Vietnam and to evaluate the factors that influence OW/OB in this population. The findings of this study will contribute to a recommendation to Vietnamese schools for an educational programme to promote healthy lifestyle behaviours.

## Methods

A cross-sectional survey was conducted in January 2014. Using a stratified sampling method, two districts were randomly selected from four urban districts in Hanoi, and then four state junior high schools were randomly selected from those two urban districts. In each school, five classes of grade six (11–12 years) were randomly selected, and all the students and their parent(s) in the selected classes were invited to participate in the study. The parents and students were required to provide written consent to participate in the study.

Each consent form required the signatures of the student and their parent(s) to enable participation; to participate, both the student and his/her parent must sign the consent form.

Students were given a self-administered questionnaire investigating their physical activity and nutrition (detail of the questionnaires in Additional file 1). They completed the questionnaires while sitting in their own classrooms and had 30 min to answer all the questions. Beforehand, public health professionals explained the details of the questionnaire and the purpose of the survey to the students.

Health examination and anthropometric measurements: Two groups, each including two doctors and three nutritionists, measured the weight of each student using BS-150WT digital weighing scales (Dretec, Koshigaya, Japan), which are precise to within 100 g [12]. Students were requested to remove their shoes and all heavy clothing. Height measurements were performed using standard metres with a precision of 0.1 cm; the metres were placed vertically, i.e., perpendicular to the ground. Students removed their sandals or shoes and then stood with their back against the metre while looking straight ahead and keeping their hands at their sides. An inelastic tape was used to measure the circumferences of the waist and hips. The results of height and circumferential measurements were recorded in centimetres to one decimal place [12]. Arterial blood pressure was measured using HEM–7051 electronic sphygmomanometers (Omron, Kyoto, Japan) [13].

Body mass index (BMI)-for-age was calculated using the formula m/h^2^, and BMI was categorized following the WHO guidelines for BMI in children aged between 5 and 19 years [14].

Information on parental anthropometric measurements, family history, and environmental factors was collected by distributing questionnaires to the students’ parent(s) (see Additional file 2). Because each student who participated in the study was weighed using BS-150WT digital weighing scales (Dretec), the same weighing scales were also used to measure the parents’ weights. To ensure the same tool measured weight and to benefit the participants of the study, all students were provided a BS-150WT digital scale. The research group provided instructions on adhesive-backed papers to instruct parents about the correct way to measure their height. The data were then recorded in the questionnaires by the students’ parent(s). Parents who had a BMI ≧25 kg/m^2^ were considered to have OW/OB based on the WHO standard cut-off points [15].

### Biochemical data

Laboratory tests were conducted in the Biochemistry Department of Bach Mai Hospital, Hanoi, Vietnam according to the ISO 15189 standard.

Students were instructed to skip breakfast on the day of the examination and had to fast for at least 10 h. The adequacy of the fasting period was confirmed before blood samples were collected. Venous blood (5 ml/student) was collected between 7:30–10:00 AM by nurses who were working in the Pediatric Department of Bach Mai Hospital in 1 g/L tubes containing ethylenediaminetetraacetic acid as an anticoagulant, and the samples were placed on dry ice. The samples were then transported from the study site to the Biochemistry Department of Bach Mai Hospital. They were centrifuged at 5000 rpm for 10 min. Plasma glucose was determined using the glucose hexokinase method with a Cobas® 8000 (c702) chemistry analyser (Roche, Basel, Switzerland). HbA1C was measured using the boronate affinity high-performance liquid chromatography method with an Ultra^2^ chromatograph (Primus Diagnostics, Kansas City, MO, USA). Insulin was determined using an electrochemiluminescence immunoassay with a Cobas® 8000 (e170) chemistry analyser (Roche). Triglycerides, cholesterol, high-density lipoprotein, and low-density lipoprotein were measured using an automated colorimetric enzyme assay with a Cobas® 8000 (e702) chemistry analyser (Roche).

### Statistical analysis

Data analysis was performed using SPSS statistics desktop version 21.0 media pack software (IBM, Armonk, NY, USA). We used a simple chi-square test at the α = 0.05 level of significance to test the associations of different risk factors. Variables showing significant associations with OW/OB in children were further identified using a logistic regression test to determine the odds ratio (OR) and the 95% CI. The risk factors for OW/OB were adjusted for sex, and multiple logistic regression analysis was used to determine the adjusted odds ratio (aOR) of each risk factor. In the analysis, there were two outcome variables: the OW/OB variable included the combined data of overweight and obesity, and the other variable incorporated the remaining data, which were not related to OW/OB in children, as a reference.

### Ethical considerations

The study protocol was approved by the Ethics Committee of Bach Mai Hospital, Hanoi, Vietnam with the decision letter Number 529 QD-BM on the 10th of May 2013 and the Ethics Committee of the National Center for Global Health and Medicine, Japan, with the number 1496 on the 1st of October 2013. Before the collection of questionnaire data and venous blood samples, information sheets and consent forms were distributed to parents and students by the schools. Students participating in the study agreed to provide written informed consent with written approval from their parent(s). All participants could withdraw from the study at any time without any threats or disadvantages.

## Results

From four districts in central Hanoi, two districts containing a total of 29 state schools were selected. Four randomly selected schools agreed to participate in the study. Information sheets and consent forms were distributed to 936 students and their parents. After both the student and his/her parent signed the consent form, 821 students participated in the study. Table 1 shows the characteristics of the students. The proportions of the students in each BMI range were evaluated according to the WHO standards. The proportion of underweight children was the lowest (3.9% of boys and 4.3% of girls). The proportions of boys (18.9%) and girls (15.4%) who were overweight did not differ significantly, χ^2^ = 1.79, *p* = 0.18. However, the proportion of obese boys (32.4%) was approximately four times higher than that of obese girls (7.7%).

**Table 1:** Prevalence for boys and girls

Proportions of boys and girls in each morphological category (based on BMI measurements)					
	Underweight % (n)	Normal % (n)	Overweight % (n)	Obesity % (n)	Total (n)
Boys (*n* = 380)	3.9% (15)	44.7% (170)	18.9% (72)	32.4% (123)	380
Girls (*n* = 441)	4.3% (19)	72.6% (320)	15.4% (68)	7.7% (34)	441
Total	4.1% (34)	59.7% (490)	17.1% (140)	19.1% (157)	821

*P* <  0.001; chi square = 91.555

Boys/girls presenting overweight or obesity: OR: 3.503 (95% CI: 2.60–4.73)

When comparing the measurements of BMI and waist circumference, it is apparent that there was a large gender difference in waist circumference (boys: 71.01 ± 9.60 cm, girls: 66.13 ± 7.62 cm, *p* = 0.001) as well as in BMI (boys: 19.9 ± 3.6 kg/m^2^, girls: 18.49 ± 2.87 kg/m^2^, *p* = 0.001).

As shown in Table 2, the biochemical data for total cholesterol and low-density lipoprotein also showed significant differences between boys and girls (total cholesterol: boys 4.33 ± 0.78 mmol/L, girls 4.20 ± 0.72 mmol/L, *p* = 0.012; LDL boys 2.37 ± 0.71 mmol/L, girls 2.20 ± 0.66, *p* = 0.001 mmol/L). Considering the family and children characteristics shown in Table 3, the odds of OW/OB were lowest among children whose parents had college / university degrees [father (aOR =0.65, 95% CI: 0.42–1.00); mother (aOR =0.63, 95% CI: 0.41–0.97)] compared with those whose parents had only a primary education. Children with an OW/OB family history had an increased risk of OW/OB. The odds were highest among children with parents with OW/OB [father (aOR =2.022, 95% CI: 1.34–3.04); mother (aOR =2.83, 95% CI: 1.51–5.30)] compared with children whose parents had a normal weight. The associated factors included parental OW/OB, and the risk was the highest among children with both parents having overweight or obesity [both parents (aOR =6.59, 95% CI: 1.28–33.87); one parent (aOR =2.22, 95% CI: 1.50–3.27)] compared with those with neither parent having overweight or obesity. Regarding birth weight (BW), the odds were greatest for high-birth-weight children [children with a BW 3500 g or greater (aOR =1.52, 95% CI: 1.07–2.15)] compared with those with a normal BW.

**Table 2:** Anthropometric and laboratory data for boys and girls

Factor	Sex	*n*	Mean ± SD	*P*
BMI (kg/m2)	boys	380	19.90 ± 3.60	0.001
	girls	441	18.44 ± 2.68	
Arm circumference (cm)	boys	376	23.51 ± 3.19	0.001
	girls	431	22.40 ± 2.32	
Hip circumference (cm)	boys	379	81.67 ± 7.89	
	girls	440	80.99 ± 6.83	0.192
Waist circumference (cm)	boys	379	71.01 ± 9.60	0.001
	girls	440	66.13 ± 7.62	
Diastolic blood pressure (mmHg)	boys	379	70.48 ± 10	
	girls	440	70.72 ± 8.6	0.567
Systolic blood pressure (mmHg)	boys	379	111.09 ± 10.3	
	girls	440	108.89 ± 12.4	0.170
Glucose (mmol/L)	boys	380	4.85 ± 0.52	0.048
	girls	441	4.78 ± 0.53	
T_cholesterol (mmol/L)	boys	380	4.33 ± 0.78	0.012
	girls	441	4.20 ± 0.72	
Triglyceride (mmol/L)	boys	380	1.05 ± 0.60	0.132
	girls	441	1.11 ± 0.54	
HDL_C (mmol/L)	boys	380	1.50 ± 0.39	0.907
	girls	441	1.49 ± 0.40	
LDL_C (mmol/L)	boys	380	2.37 ± 0.71	0.001
	girls	441	2.20 ± 0.66	
HbA1c (%)	boys	380	5.44 ± 0.26	0.362
	girls	441	5.42 ± 0.24

**Table 3:** Factors associated with overweight or obesity among children aged 11–12 years, Hanoi, Vietnam

Factor	OR	95% CI		*p*	aOR	95% CI		*p*
		Lower	Upper			Lower	Upper	
FAMILY CHARACTERISTICS								
Father’s education								
Primary school	1				1			
Secondary school	0.80	0.15	4.23	0.801	0.72	0.12	4.32	0.715
College or university	0.73	0.48	1.09	0.122	0.65	0.42	1.00	0.050
Mother’s education								
Primary school	1				1			
Secondary school	1.62	0.46	5.68	0.448	1.14	0.30	4.31	0.842
College or university	0.69	0.46	1.04	0.079	0.63	0.41	0.97	0.037
Father’s BMI								
18.5–24.9	1				1			
< 18.5	0.66	0.29	1.45	0.317	0.62	0.26	1.44	0.265
≥ 25	1.92	1.30	2.84	0.001	2.02	1.34	3.04	< 0.001
Mother’s BMI								
18.5–24.9	1				1			
< 18.5	0.68	0.28	1.63	0.368	0.69	0.28	1.72	0.428
≥ 25	2.28	1.26	4.13	0.007	2.83	1.51	5.30	0.001
Parental overweight or obesity (classified by BMI)								
Neither parent overweight or obese	1				1			
One parent overweight or obese	1.94	1.35	2.80	0.001	2.22	1.50	3.27	< 0.001
Both parents overweight or obese	7.18	1.50	34.89	0.015	6.59	1.28	33.87	0.024
CHILD CHARACTERISTICS								
Number of siblings in the family								
1	1				1			
2	1.27	0.79	2.04	0.323	1.29	0.79	2.11	0.305
≥ 3	0.66	0.33	1.26	0.202	0.87	0.43	1.75	0.697
Birth order among siblings								
1	1				1			
2	0.89	0.66	1.19	0.420	0.80	0.58	1.09	0.149
≥ 3	0.40	0.16	1.00	0.050	0.47	0.18	1.22	0.122
Breastfeeding history (none as reference)								
Breastfed by mother for at least 6 months after birth	0.80	0.58	1.12	0.191	0.87	0.62	1.22	0.410
Birth weight (grams)								
2500–3500	1				1			
< 2500	0.40	0.18	0.87	0.021	0.51	0.22	1.14	0.101
≥ 3500	1.60	1.14	2.23	0.006	1.52	1.07	2.15	0.019
Time spent watching television (hours per day)								
< 2	1				1			
2–4	0.97	0.67	1.41	0.862	1.00	0.68	1.49	0.989
> 4	1.56	0.89	2.72	0.122	1.78	0.99	3.20	0.056
Sleep per day (hours)								
< 8	1				1.00			
8–11	0.64	0.46	0.90	0.009	0.57	0.40	0.81	< 0.001
> 11	0.47	0.24	0.90	0.022	0.44	0.22	0.87	0.018
PHYSICAL ACTIVITY AND LIFESTYLE BEHAVIOURS OF CHILDREN								
Exercised in last 7 days (0–2 days/week as reference compared with those who did 3 days or more per week)								
Exercised for 60 min per day	1.19	0.87	1.63	0.276	0.96	0.69	1.34	0.83
Intense exercise for 20 min per day evidenced by sweating and breathing hard	1.15	0.82	1.62	0.411	0.97	0.68	1.37	0.84
Participation in sports in the last 12 months (None as reference)	1.14	0.48	2.7	0.765	1.22	0.49	2.98	0.67
Lifestyle behaviours (None as reference)								
Weight-reducing exercises	0.17	0.12	0.23	< 0.001	0.16	0.11	0.23	< 0.001
Lowering food intake	0.45	0.11	0.20	< 0.001	0.12	0.09	0.17	< 0.001
Adding vegetables to diet	0.26	0.19	0.36	< 0.001	0.26	0.19	0.35	< 0.001

Odds ratio (OR) by logistic regression univariate analysis

Ajusted OR by multivariate logistic regression analysis by each individual variable and controled for sex

Regarding length of sleep, the odds of OW/OB were lowest among children who slept more than 11 h per day [sleeping 8–11 h (aOR =0.57, 95% CI: 0.40–0.81)], followed by those who slept between 8 and 11 h [(aOR =0.44, 95% CI: 0.22–0.87)] compared with those who slept less than 8 h.

Children with specific positive lifestyle behaviours had lower risks for OW/OB. Furthermore, the odds of developing OW/OB was lower among children who exercised for weight reduction (aOR =0.16, 95% CI: 0.11–0.23), lowered food intake (aOR = 0.12, 95% CI: 0.09–0.17), and added vegetables to their diet (aOR = 0.26, 95% CI: 0.19–0.35).

## Discussion

The study identified the prevalences of overweight (17.1%) and obesity (19.1%) among schoolchildren aged 11–12 years in central Hanoi from a survey conducted in 2014, and the prevalences were higher than the national averages. A prior survey of the nutritional status of children in primary schools within urban areas of Hanoi was conducted by the Vietnam National Institute of Nutrition in 2011. That previous survey included participants aged 7–9 years and found rates of overweight and obesity of 23.4 and 17.3%, respectively [16]. Another study in 2010 that included adolescents aged 11–14 years old in Ho Chi Minh City found rates of overweight and obesity of 19.6 and 7.9%, respectively [17]. Obesity is a risk factor for NCDs, and Vietnam has been identified as a country with a major problems associated with NCDs, which are responsible for approximately 73% of total deaths [5]. There are several studies about the risk factors for OW/OB in Vietnam. A study performed in central Vietnam showed that BMI was the largest contributor to diabetes mellitus risk, with an OR of more than ten times compared to low- and normal-BMIs [18]. Therefore, preventing OW/OB in children plays an important role in preventing NCDs in the future.

In addition to determining the prevalence of OW/OB, our study resulted in several other findings. Children of parents with college or university degrees had the lowest risks for OW/OB. Our result was concordant with those of previous studies. Many studies in developed countries, such as the USA, Brazil, France, Denmark, and Appalachia, showed similar results as ours [19–22]. However, there were opposite tendencies in Colombia and Kenya, where maternal education was positively related to child overweight and negatively associated with physical activity among children. The researchers suggested that there was a possibility that such relationships might be related to the developmental stage of different countries [19].. Antonogeorgos G, et al. suggested that parental education status seemed to play a mediating role in the beneficial effect of diet on children’s obesity status [19]. Our results suggested that education for parents could be effective for reducing OW/OB among children in Vietnam.

We also found that parental OW/OB is a risk factor for OW/OB among children. This result is similar to the results of previous studies conducted in South Korea from 2007 to 2010 [23] and in the UK from 1998 to 2001 [24]. The study in the USA showed that among children, especially older children, obesity was an increasingly important predictor of adult obesity [6]. Therefore, it is vital to create a strategy for preventing OW/OB among children who have parents with OW/OB. Those parents and children should emphasize healthy dietary habits and regular exercise from an early age. The study in the USA also showed that parental obesity was a risk factor for adult obesity among both obese and non-obese children [6]. A suitable educational programme should be implemented within schools as well as in communities to reduce the burden of OW/OB among children and adults in the future.

Several studies have shown that breastfeeding contributes to preventing OW/OB in children [25–27]. Our study showed a similar trend; however, this finding did not reach statistical significance. One study from Scotland in 1999 reported that there was no association between breastfeeding and OW/OB among children [28].

According to a meta-analysis, most studies have concluded that BW is a factor that contributes to OW/OB [29, 30]. A cohort study from Sicily in 2016 also supported this association [31]. Our present study additionally found that a BW of 3500 g or more was significantly positively associated with OW/OB in Vietnamese children. A meta-analysis reported that pre-pregnancy OW/OB in women increased the risk of high birth weight (HBW) and subsequent offspring OW/OB [31]. In addition, there have been several trials of interventions to reduce the risk of OW/OB in infancy and early childhood [31]. We suggest that an educational programme on weight management for pregnant women be conducted to prevent OW/OB among children.

Many studies have shown that time spent watching television is associated with an increased risk of OW/OB [32–35]. Our study also found a trend towards an increased risk of OW/OB among children who watched television for longer than 4 h per day, but this finding hardly reached statistical significance. It was suggested that time spent watching TV might be related to several mechanisms leading to OW/OB among children, such as decreased physical activity, increased energy intake, increased sedentary behaviour, exposure to food advertising and reduced sleep time [32–35]. Further investigation is necessary to clarify such relationships for the intervention of OW/OB among Vietnamese children.

The results of the study showed that the duration of sleeping was negatively associated with OW/OB among children. This is the same result as that of a meta-analysis [36]. The data from a study in Portugal showed that the OR for childhood obesity decreased with sleep duration (reference 8 hours/day (h/d); 9–10 h/d: 0.44; ≧11 h/d: 0.39) [37]. A meta-analysis of 12 studies including 15 cohorts showed that short sleep duration was significantly associated with obesity (relative risk 1.30, 95% CI: 1.20–1.42), even after the exclusion of 2 cohorts that substantially affected the heterogeneity [36]. Shorter night-time sleep could be associated with higher total energy intake [38] and less physical activity [39]. However, the precise mechanisms underlying the association between short sleep duration and obesity have not been elucidated. It was suggested that improvement in child sleep duration would have a positive impact on OW/OB among children [40].

We evaluated self-perceived assessment of health-promoting behaviours for lifestyle improvement among children. We obtained one important finding that self-efficacy of specific positive lifestyle behaviours was strongly associated with reduced risks of OW/OB among children. There are several studies reporting the associations between self-efficacy and obesity among children [41–46]. One study reported that there were no differences in physical activity between normal-fat and over-fat children [41]. However, another study in Korea reported that there were differences in eating habits, physical activity, and self-efficacy between children with normal weight and OW/OB [42]. The researchers suggested that the increase in self-efficacy be one of the targets for obesity management programmes among children. It was reported that perceived physical self-efficacy and BMI were negatively associated among schoolchildren, suggesting the importance of the psychological aspect of obesity [43]. Another study reported that there were significant differences in healthy-eating-specific self-efficacy levels between students of normal weight and those with OW/OB [44]. Additionally, children with high self-efficacy were reported to participate in significantly more physical activity than those with low self-efficacy [41]. In fact, there have been several intervention studies for the treatment of OW/OB in children or adolescents [45, 46]. Several studies have suggested that parent and community involvement was effective in reducing the risk of obesity in schoolchildren [47–50]. At the ages of 11–12, children become capable of making independent decisions regarding their diet and can exercise to keep their body healthy. We think that this age range is the right time to start treating OW/OB in children. Further investigation is needed to clarify the factors associated with self-efficacy and OW/OB in children or adolescents to reduce the risk of obesity in children.

### Limitations

This study has some limitations regarding the generalizability of our findings. First, this study was a cross-sectional study. Therefore, we could not address causal relationships of variables or factors related to obesity. The potential causal relationships must be clarified by longitudinal studies. Second, this study was limited to the urban area of Hanoi, and the results may not be applicable to all populations in Hanoi; however, the results could be a reference for large cities in Vietnam, such as Ho Chi Minh, Da Nang, and Hue cities. Further studies should include students in other areas with a focus on identifying lifestyle factors that can effectively prevent and treat OW/OB. Third, we did not investigate the factors associated with our findings. We found that children of parents with college or university degrees had the lowest risks for OW/OB and that children who showed specific positive lifestyle behaviours had reduced risks of OW/OB. In the future, we need to conduct further investigations to clarify specific associated factors related to these findings. Finally, an effective educational programme should be implemented in schools to improve students’ knowledge and induce behavioural changes that can contribute to reducing the prevalence of OW/OB among children in urban and rural areas of Vietnam based on our findings.

## Conclusion

The prevalences of OW/OB among 11- to 12-year-old students in central Hanoi were quite high compared with the data from other studies in Hanoi and Ho Chi Minh City in Vietnam [16, 17]. This study also found that several characteristics of the children, their families, and their lifestyle behaviours were strongly associated with the risks of OW/OB. The results suggested that parental education level might be associated with OW/OB among children and that families with parents with OW/OB and children with a high BW should be educated to prevent OW/OB at an early age. In addition, it is suggested that to reduce the prevalence of OW/OB among children, targets for positive lifestyle changes should be set and managed by the students. Further studies should focus on the intervention and treatment of OW/OB among schoolchildren aged 11–12 years.

## Supplementary information

**Additional file 1.** Survey Questionnaire (for students).

**Additional file 2.** Questionnaire for parent.

## Data Availability

The datasets generated and/or analysed during the current study are not publicly available but are available from the corresponding author on reasonable request.

## Abbreviations

BMI: Body mass index
BW: Birth weight
CI: Confidence interval
ISO: International Organization for Standardization
NCDs: Non-communicable diseases
OR: Odds ratio
OW/OB: Overweight and obesity
WHO: World Health Organization
