# Supplementary material for: Prevalence and associated factors of overweight and obesity among schoolchildren in Hanoi, Vietnam
Source: BMC Public Health. 2019 Nov 8;19:1478. doi: 10.1186/s12889-019-7823-9 (PMC6839165; doi:10.1186/s12889-019-7823-9)
Supplement: Supplementary file 1 — Additional file 1. Survey Questionnaire (for students). [file 12889_2019_7823_MOESM1_ESM.docx]

**SURVEY QUESTIONNAIRE**

**(for students)**

| 1. **General information** | | | | | | | |
| --- | --- | --- | --- | --- | --- | --- | --- |
| 1 | What’s your date of birth? | | | | | | ……./……/………. |
| 2 | What’s your sex? | | | | | | 1. boy 2. girl |
| 1. **Physical and sedentary activities questions** | | | | | | | |
| 3 | | **Yesterday,** were you physically active for a total of **at least 60 minutes**? (Add up all the time you spent in any kinds of physical activity that increased your heart rate and made you breathe harder.) | | | | | |
|  |  | 1. Yes | | | | 1. No | |
| 4 | | During the **past 7 days**, on how many days were you physically active for a total of at least 60 minutes per day? …..day | | | | | |
| 5 | | On how many of the past 7 days did you exercise or participate in high-intensity physical activities for at least **20 minutes that made you sweat and breathe hard** (for example: tennis, football, martial arts, shuttle cock, rope skipping, aerobics, running…)? ………day | | | | | |
| 6 | | How many hours do you spend time **for video, games and other activities on computers, iPads and mobile phones** which is not school work? | | | | | |
|  |  | 1. No, I don’t. 2. Less than 1 hour per day 3. 1 hour per day 4. 2 hours per day | | | | 1. 3 hours per day 2. 4 hours per day 3. 5 or more hours per day | |
| 7 | | On average, on how many hours do you spend for watching TV a day? …hours | | | | | |
| 8 | | How far is it from your house to your school?............................(km) | | | | | |
| 9 | | How do you go to school? | | | | | |
|  |  | 1. On foot 2. Bike 3. Motorbike | | | | 1. Bus 2. Car 3. Other……………………. | |
| 10 | | In an average day when you are at home, how much time do you spend **doing housework** (such as dishwashing, house wiping, parching, cooking, babysitting)? | | | | | |
|  |  | 1. I don’t 2. Less than 1h per day 3. 1-2 hours per day | | | | 1. 2 -3 hours per day 2. Over 3 hours per day | |
| 11 | | On average how much time do you spend **sleeping** per day? | | | | | |
|  |  | 1. I don’t know 2. Less than 6 hours per day | | | | 1. 6-8 hours per day 2. Over 8h per day | |
| 12 | | In the evening, what time do you go to bed? ………… ………..  In the morning what time do you wake up? ............ ............... | | | | | |
| 13 | | Do you have noon naps? 1.Yes …………… 2. No………………….. | | | | | |
| 14 | | If yes, on average how much time do you spend on naps per day? .............minutes | | | | | |
| 15 | | During the past 12 months, how many kinds of sports did you play? (Include teams run by your school or community groups.) | | | | | |
|  |  | 1. 0 teams 2. 1 team | | | | 1. 2 teams 2. 3 or more teams | |
| For each of the following activities, how many day you did the activity during the past 12 months? | | | | | | | |
| Activity | | | Yes/No | | Number of days in past 7 days | | |
| 16. Football | | | Yes/No | | 0 1 2 3 4 5 6 7 | | |
| 17. Badminton | | | Yes/No | | 0 1 2 3 4 5 6 7 | | |
| 18. Kick a shuttle cock | | | Yes/No | | 0 1 2 3 4 5 6 7 | | |
| 19. Aerobics | | | Yes/No | | 0 1 2 3 4 5 6 7 | | |
| 20. Dance sport | | | Yes/No | | 0 1 2 3 4 5 6 7 | | |
| 21. Swimming | | | Yes/No | | 0 1 2 3 4 5 6 7 | | |
| 22. Bike riding | | | Yes/No | | 0 1 2 3 4 5 6 7 | | |
| 23. Running | | | Yes/No | | 0 1 2 3 4 5 6 7 | | |
| 24. Walking | | | Yes/No | | 0 1 2 3 4 5 6 7 | | |
| 25. Volleyball | | | Yes/No | | 0 1 2 3 4 5 6 7 | | |
| 26. Basketball | | | Yes/No | | 0 1 2 3 4 5 6 7 | | |
| 27. Martial arts | | | Yes/No | | 0 1 2 3 4 5 6 7 | | |
| 28. Tumbling | | | Yes/No | | 0 1 2 3 4 5 6 7 | | |
| 29.Tracks and field | | | Yes/No | | 0 1 2 3 4 5 6 7 | | |
| 30. Others (list in detail) | | | Yes/No | | 0 1 2 3 4 5 6 7 | | |
| 1. **Nutrition and body weight questions** | | | | | | | |
| 31 | On average, **how many meals** do you have per day ? | | | | | | |
| 32 | During the past 7 days, on how many days did you **not eat breakfast**? | | | | | | |
| 33 | During the past 7 days, on how many days did you not have **lunch?** | | | | | | |
| 34 | How many of the past 7 days did you not eat **dinner**? | | | | | | |
| 35 | Which of the following are you trying to do about your weight during the past 30 days? | | | | | | |
|  | 1. Lose weight 2. Gain weight | | | 1. Stay the same weight 2. I’m not trying to do anything | | | |
| 36 | During the past 30 days, did you do exercise to lose weight? | | | | | | |
|  | 1. Yes | | | 1. No | | | |
| 37 | During the past 30 days, did you lower your food intake to lose weight? | | | | | | |
|  | 1. Yes | | | 1. No | | | |
| 38 | During the past 30 days, did you eat more vegetables to lose weight? | | | | | | |
|  | 1. Yes | | | 1. No | | | |
