# Supplementary material for: Prevalence and associated factors of overweight and obesity among schoolchildren in Hanoi, Vietnam
Source: BMC Public Health. 2019 Nov 8;19:1478. doi: 10.1186/s12889-019-7823-9 (PMC6839165; doi:10.1186/s12889-019-7823-9)
Supplement: Supplementary file 2 — Additional file 2. Questionnaire for parent. [file 12889_2019_7823_MOESM2_ESM.docx]

**Questionnaire for parent**

| 1 | Commune ………………… District………………... |
| --- | --- |
| 2 | Year of birth: Mother ……………… Father ………… |
| 3 | Parents’ education level   \| Mother \| Father \| \| --- \| --- \| \| 1. Elementary. 2. Secondary school. 3. High school. 4. University and over. \| 1. Elementary. 2. Secondary school. 3. High school. 4. University and over. \| |
| 4 | Parents’ occupation   \| Mother ……………………. \| Father ………………….. \| \| --- \| --- \| |
| 5 | Parents’ weight (in kilograms)   \| Mother ……………………. \| Father ………………….. \| \| --- \| --- \| |
| 6 | Parents’ height (in centimeters)   \| Mother ……………………. \| Father ………………….. \| \| --- \| --- \| |
| 7 | How many children do you have?................... |
| 8 | What is the order of your child (participating in this research) among their siblings? |
| 9 | Birth weight …….. grams |
| 10 | Was your child breastfed in their first 6 months of age?   1. Yes 2. No |
